# High‐Speed 3D Printing Coupled with Machine Learning to Accelerate Alloy Development for Additive Manufacturing

**DOI:** 10.1002/advs.202414880

**Published:** 2025-03-07

**Authors:** Avinash Hariharan, Marc Ackermann, Stephan Koss, Ali Khosravani, Johannes Henrich Schleifenbaum, Patrick Köhnen, Surya R. Kalidindi, Christian Haase

**Affiliations:** ^1^ Steel Institute RWTH Aachen University 52072 Aachen Germany; ^2^ Salzgitter Mannesmann Forschung GmbH 38239 Salzgitter Germany; ^3^ Chair of Digital Additive Production RWTH Aachen Aachen Germany; ^4^ Woodruff School of Mechanical Engineering Georgia Institute of Technology Atlanta 30332 USA; ^5^ Multiscale Technologies llc Atlanta 30308 USA; ^6^ GKN Additive 53117 Bonn Germany; ^7^ Chair Materials for Additive Manufacturing Technical University Berlin D‐10587 Berlin Germany; ^8^ Center for 3D Technologies Technical University Berlin D‐10623 Berlin Berlin Germany

**Keywords:** alloy design for AM, alloys for additive manufacturing, high‐speed DED, high‐throughput screening, machine learning

## Abstract

Developing novel alloys for 3D printing of metals is a time‐ and resource‐intensive challenge. High‐throughput 3D printing and material characterization protocols are used in this work to rapidly screen a wide range of chemical compositions and processing conditions. In situ, alloying of high‐strength steel with pure Al in the targeted range of 0–10 wt.% and flexible adjustment of the volumetric energy input is performed to derive 20 individual alloy combinations. These conditions are characterized using large‐area crystallographic analysis combined with chemistry and nanoindentation protocols. The significant influence of Al content and processing conditions on the constitutive material behavior of the metastable base alloy allowed for efficient exploration of the underlying process‐structure‐properties (PSP) relationships. The extracted PSP relations are discussed based on the dominant physical mechanisms observed in the samples. Furthermore, the microstructure‐property relationship based on limited experimental data is supported by an explainable machine‐learning approach.

## Graphical abstract

This work shows combining high‐throughput experiments of metal 3D printing with data science‐driven prediction leads to faster alloy development. Researchers develop steel alloy specimens specifically for 3D printing by adjusting aluminum content (0–10 wt.%) and energy input during printing. Through crystallographic, chemical, and mechanical analysis, key relationships between processing conditions, microstructure, and properties, enabling machine learning models are identified to predict alloy behavior efficiently.

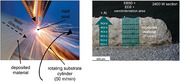

## 1 Introduction

Additive manufacturing (AM) stands out as a highly promising technology for sustainable production and design‐specific fabrication of high‐performance components.^[^ ^1^ ^]^ Notably, the metal AM sector is anticipated to experience substantial growth in the coming years.^[^ ^2^ ^]^ While AM machines have reached nominal levels of industrial maturity, there remains a significant limitation in their application to a wide range of metallic alloys. Among the limited alloys that can be produced via AM, only a very small portion was specifically designed for AM.^[^ ^2^, ^3^, ^4^, ^5^, ^6^ ^]^ The primary challenge hindering the widespread use of AM techniques is the complexity of optimizing the extensive accessible parameter space while obtaining defect‐free specimens. Success in this endeavor is inherently dependent on the response of conventional alloys to the rapid solidification process. Moreover, rapid solidification conditions during AM are being leveraged to design new alloys and develop novel microstructures.^[^ ^6^ ^]^ The parameter space for designing new alloys for/and using AM is high‐dimensional, including features describing the raw materials (such as chemical composition, size, morphology of the powder, powder production techniques, and their associated parameters) and AM processing parameters (including laser power, angle, raster pattern, and spacing), and post‐AM processing (such as annealing). It is essential to implement high‐throughput screening protocols in all aspects of the AM process development: (i) the fabrication process, (ii) the microstructure characterization, and (iii) the mechanical property assessment to expedite microstructure and alloy development.

Laser‐based powder bed fusion of metals (PBF‐LB/M) is the leading AM process in industries for fabricating complex‐shaped parts. However, designing new metallic alloys for PBF‐LB/M (and AM in general) presents challenges primarily due to the time‐intensive atomization process required to produce pre‐alloyed powders. This requirement greatly increases lead times for alloy design using the PBF‐LB/M process. In these circumstances, the dominant advantage of laser‐based AM techniques, Direct Energy Deposition (DED), and Extreme High‐Speed Laser Material Application (EHLA) over other methods such as Laser Powder Bed Fusion (LPBF),^[^ ^7^, ^8^ ^]^ is to create miniature alloy specimens fabricated with diverse process parameter combinations required for high‐throughput characterization protocols. These include precise control over composition (via in‐situ supply of multiple powders) and process parameters (e.g., a wide range of cooling conditions, influencing solidification behavior) of the individual layers, resulting in a wide range of microstructures of the printed specimens. Although previous studies, focusing on AM alloy compositions for steels,^[^ ^9^ ^]^ nickel alloys,^[^ ^10^ ^]^ and high‐entropy alloys,^[^ ^11^ ^]^ involve the use of DED, the transferability of this knowledge to LBPF is challenging due to its contrasting process and solidification conditions. In this regard, the EHLA process allows for efficient, cost‐effective, and easy manipulation of the process parameters that overlap the domains of DED and LPBF solidification conditions. Coupled with a wide range of laser scanning speeds up to 200 m min^−1^, the typical cooling rates observed in the EHLA process range between 10^4^ to 10^6^ K s^−1^, overlapping with the conditions observed in DED and LPBF respectively. Therefore, EHLA offers an opportunity to simulate process conditions and cooling rates across a range from DED to LPBF, offering control over the solidification cell structure, phase fraction, and other microstructural features that mimic the desired range of processing conditions.^[^ ^12^, ^13^ ^]^ This aids in high‐throughput design of novel alloys via in‐situ powder mixing to rapidly screen microstructures and mechanical properties. The traditional EHLA process, normally utilized in coating technologies, has been explored here for the first time to evaluate its potential for miniature sample production for rapid exploration of AM‐specific alloy design.

The highly nonlinear mappings between the AM process parameters and the material properties of interest in the produced parts have prompted the use of data‐driven approaches for capturing the important relationships required to accelerate alloy design. The integrated computational materials engineering approach (ICME), incorporating physics‐based tools to formulate the desired process‐structure (P‐S)^[^ ^14^, ^15^ ^]^ and structure‐property (S‐P)^[^ ^16^ ^]^ relationships, computationally expensive and time‐consuming. The previously established experimental and ICME‐based approaches in the materials science field can be enhanced using machine learning (ML) algorithms to accelerate AM alloy design. Most prior ML‐assisted experimental approaches have focused on component design for AM, AM process monitoring,^[^ ^17^, ^18^ ^]^ and AM digital production. The potential to exploit these techniques in accelerated alloy design with P‐S and S‐P linkages has been less explored.^[^ ^19^ ^]^ Popova et al.^[^ ^15^ ^]^ primarily established a general automated workflow to extract P‐S linkages for synthetic AM microstructural evolution. The primary challenge arises from the limited data size and the high expense of producing large data sets (from both experiments and simulations). Ackermann et al.^[^ ^19^ ^]^ elucidated a computationally efficient data‐driven framework achieving nearly 91% accuracy in predictive quantification of P‐S linkages with 960 synthetically generated 3D microstructures mimicking powder bed fusion. The study revealed how ML‐based approaches can be applied to identify desired microstructures within the wide process‐parameter space.

In this work, we explore the benefits of an EHLA‐based high‐throughput screening protocol across the entire cycle, encompassing fabrication, microstructure quantification, property measurements, and data acquisition for training ML‐based surrogate models. Toward this goal, high manganese steel (HMnS) samples with ≈21 wt.% Mn is fabricated, with varying aluminum content (in the range of 0–10 wt.%), via in‐situ mixing of gas‐atomized powders of high manganese steel and pure aluminum. HMnS is chosen here as a promising candidate due to its inherent work‐hardenability by the activation of different deformation mechanisms, through the manipulation of stacking fault energy (SFE) and phase composition of fcc and bcc phases with targeted additions of Al. Alloying additions to HMnS can stabilize or destabilize the austenite (fcc phase) and secondly, promote the formation of a single‐phase or dual‐phase microstructure with varied deformation mechanisms. Aluminum additions to HMnS lower the density of high Mn steels, reduce strength ductility trade‐off, enhance their oxidation resistance due to the formation of an Al_2_O_3_‐rich protective layer, and wear resistance because of their high/work hardening capacity.^[^ ^2^, ^20^, ^21^, ^22^ ^]^

This alloy design concept coupled with rapid solidification conditions of AM is used to tune the stability, and fraction of fcc austenite and bcc ferrite to obtain a wide range of mechanical responses for structural parts. The maximum Al addition is restricted to 10 wt.%, as a further increase can render the alloy susceptible to solidification cracking and cavitation.^[^ ^23^ ^]^ Furthermore, our previous studies using LPBF^[^ ^24^, ^25^ ^]^ and DED^[^ ^25^ ^]^ of HMnS + (0–2 wt.%) Al alloy revealed differences in segregation of solutes, solidification and melt pool characteristics, and grain size differences. This understanding provides a strong fundamental background to compare with the proposed novel approaches using the EHLA process. The microstructure of the EHLA fabricated samples is assessed using Electron Backscatter Diffraction (EBSD) combined with energy‐dispersive X‐ray Spectroscopy (EDS). Additionally, mechanical properties are evaluated utilizing spherical nanoindentation stress‐strain curve protocols on sample surfaces without the need for producing macroscopic samples for mechanical testing (e.g., tensile specimens). From the experimental EBSD data, suitable grain descriptors were extracted, and the yield strength derived from nanoindentation experiments was benchmarked as the target. With this understanding, a specific parameter set from EHLA was translated to the LPBF process to fabricate alloys of similar chemical composition, to study their macroscopic mechanical response using tensile tests. The results are compared with the data obtained from the EHLA samples. The overall approach presented here is generalizable to a broad range of AM metals to further advance the field of additive manufacturing in the direction of a sustainable manufacturing process.

## 2 Experimental Section

### 2.1 Material and Methods

#### 2.1.1 Powders for EHLA Process

X30Mn21 powder, supplied by ThyssenKrupp Raw Materials GmbH (Germany), was produced via argon gas atomization using the EIGA (Electrode Induction Melting Gas Atomization) technique. Likewise, Al powder, provided by TLS Technik GmbH (Germany), underwent argon gas atomization through the VIGA (Vacuum Induction Melting Gas Atomization) technique. The chemical compositions of both powder materials are presented in **Table** **1**. The powder particles were spheroidal in shape and underwent sieving and air separation to achieve a size distribution ranging from 10 to 45 µm. The particle size distribution was determined through optical image analysis following ISO 13322‐2, utilizing a Camsizer X2 particle analyzer (Retch Technology GmbH, Germany). To improve mixability with the larger X30Mn21 powder particles, the Al powder size distribution was adjusted toward smaller particle sizes. The average particle size was measured at 14.94 µm, with the first decile (D10) at 9.95 µm, the median (D50) at 12.99 µm, and the last decile (D90) at 22.84 µm. The apparent powder density was found to be 4.11 g/cm^3^, as measured using the Hall funnel method per ISO 3923‐1. Additionally, the avalanche angle was recorded at 35.53° using a revolution powder analyzer (PS Prozesstechnik GmbH, Germany).

**Table 1:** Main process parameters used for the EHLA process.

Laser power [W]	Track displacement [mm per rev]	Process speed [m min^−1^)	Powder mass flow X30Mn21 [g min^−1^]	Powder mass flow Al [g min^−1^]	Carrier Ar gas flow [l min^−1^]	Shielding Ar gas flow [l min^−1^]	Laser spot diameter [mm]
2400–3000	0.35	50	25.63	0‐7	7	12	1.2

#### 2.1.2 Sample Fabrication by Extreme High‐Speed Laser Material Deposition

Microstructure analysis and mechanical testing specimens were 3D printed using a Hornet EHLA system (Hornet Laser Cladding BV, Netherlands). This system features a 4‐axis handling mechanism equipped with a tiltable turning spindle, enabling the processing of rotationally symmetrical components with dimensions of up to Ø 400 × 400 mm. The rotation speed is adjustable, reaching up to 650 rpm. The laser source is a TruDisc8001 disk laser (Trumpf GmbH + Co. KG., Germany) with a wavelength of 1030 nm and a maximum output power of 8 kW. Seamless precision steel tubes (E355 + C (St52‐BK), EN 10305‐1/DIN 2391) serve as the substrate cylinders for the printing process.^[^ ^12^, ^13^ ^]^

On the tubes, 7 mm wide tracks with multiple layers are applied with substrate at room temperature. Each stripe defines a different process parameter set. The process parameters used for the EHLA samples produced for this study are provided in **Table** **2**. The laser powers for the individual specimens were varied within the range of 2400 up to 3000 W while keeping the other process parameters constant.

**Table 2:** Chemical composition of X30Mn21 metal powder and pure aluminum powder. Fe, Mn, Al, Cr, and Ni were measured by ICP‐OES and C, O by the combustion method. All contents are given in wt.%.

Powder	Fe	Mn	C	Al	Cr	Ni	O
**X30Mn21**	balance	21.0	0.330	0.030	0.150	0.080	0.100
**Al**	–	–	–	balance	–	–	0.001

The particle velocity and the powder mass flow rate in the powder gas stream are the process parameters that influence the energy input into the powder particles and the substrate material. As ṁ_p_ increases, the energy absorption per powder particle decreases. This results in reduced heating of the particles. An increased carrier gas flow rate leads to an increased v_particle_ and reduced interaction time with the laser beam, thereby reducing the heating of individual particles. The energy input into the substrate is mostly influenced by process speed (v_p_) and track displacement (f) which is the lateral distance between one coating pass and the following pass.^[^ ^12^, ^13^ ^]^ With increasing v_p_, the interaction time between the laser beam and the substrate decreases, reducing energy input into the substrate. This leads to a reduction of melt pool size and the size of the heat‐affected zone. Further details of the physics behind the EHLA process and the comparison with LPBF and DED have been described in prior work.^[^ ^12^, ^26^, ^27^ ^]^

#### 2.1.3 Sample Fabrication by LPBF for Validation

Powder mixtures of X30Mn21 and Al were produced using a Turbula 2F tumbler mixer (Willy A. Bachofen AG, Switzerland) for 45 min to ensure a homogeneous distribution of powder particles. Additively manufactured bulk specimens for mechanical testing and microstructure analysis were produced using an EOS SINT 270 LPBF machine (EOS GmbH, Germany) equipped with a single‐mode fiber Yb: YAG‐laser (400 W) and a Gaussian laser intensity profile. The building chamber was flooded with argon gas (purity ≥ 99.996%) with a flow rate of 3 L mi^−1^n to achieve a build chamber excess pressure of 100 mbar and an average oxygen concentration of ≈100 ppm during LPBF. A laser power of 120 W and a volumetric energy density (Ev) of 76.19 J mm^−3^ were used in combination with a bidirectional scan strategy. Two scan rotation strategies, 0 and 90° between each layer were chosen to replicate the layer‐wise deposition in DED and LPBF respectively. These process parameters produced dense bulk specimens with a relative density of ≥ 99.9%.

#### 2.1.4 Sample Preparation

Microstructural characterization was conducted on the cross‐section of the substrate cylinder + EHLA deposited material. The specimens were prepared for EBSD analysis by mechanical cutting, mechanical grinding (up to 1200 SiC grit paper), mechanical polishing (3 and 1 µm diamond suspensions), and electro‐polishing with a LectroPol‐5 electrolytic polishing machine (Struers GmbH, Germany) using an A2 electrolyte (Struers GmbH, Germany) for 20 s at 28 V at room temperature.

#### 2.1.5 Tensile Testing of LPBF Samples

LPBF‐produced bulk specimens were subjected to uniaxial tensile testing using a Z100 testing device (Zwick/Roell GmbH & Co. KG, Germany) at a strain rate of 0.001 s^−1^ at room temperature. During tensile testing, the strain was measured with a videoXtens extensometer (Zwick/Roell GmbH & Co. KG, Germany) and the force with an Xforce load cell (Zwick/Roell GmbH & Co. KG, Germany). Three tensile specimens were tested for each condition.

#### 2.1.6 Microstructure Characterization of EHLA Specimens

For EBSD and EDS analyses, a field emission gun SEM (Carl Zeiss AG, Germany) was used in combination with a NordlysNano (Oxford Instruments plc, UK) detector with a step size of 500 nm. EBSD and EDS measurements were conducted in the region of interest (ROI) shown to examine the existing phases, average grain size, average grain aspect ratio defined as the ratio of length/width (major axis and the one perpendicular) of the grains, and the microtexture. The EBSD data were processed and analyzed with the MATLAB (Mathworks Inc., USA) toolbox MTEX.^[^ ^28^, ^29^, ^30^ ^]^ The grain size was defined as the diameter of a circle with an area equivalent to the measured grain. Smoothing of EBSD data was performed with a half‐quadratic filter, which preserves inner grain structures. High‐angle grain boundaries were defined by a misorientation angle θ ≥10° between adjacent measurement points. For combined EDS and EBSD measurements, an X‐Max 50 EDS detector (Oxford Instruments PLC, UK) was used.

#### 2.1.7 Spherical Nanoindentation Strain–Stress Analysis on EHLA Specimens

**Figure 1:**
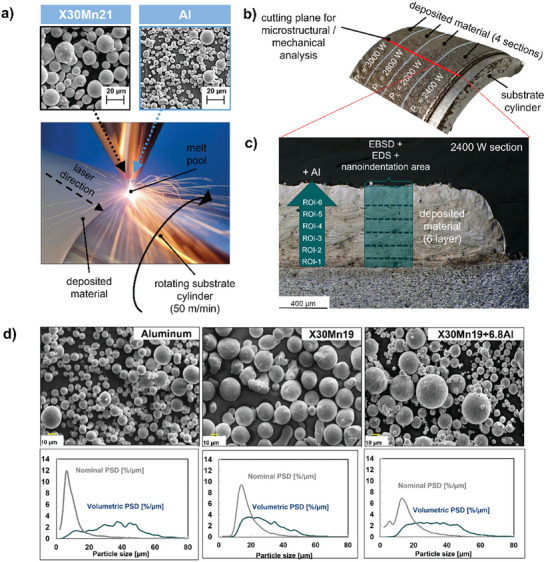
a) Illustration of the extreme high‐speed laser material application (EHLA) process used in this study. X30Mn21 and Al powder are mixed in the nozzle and deposited on a rotating substrate cylinder using a laser beam with varying laser power (2400–3000 W). b) Cut out of the substrate cylinder and deposited material with different volumetric energy densities by increasing the laser power in four neighboring (horizontal) sections of the specimen. The cutting plane for microstructural and mechanical analysis is marked as a red line. c) Example micrograph of the 2400 W section revealing 6 deposited layers with increasing Al‐content (0–8 wt.%) and the area for EBSD, EDS, and nanoindentation analysis. d) The nominal and volumetric size distribution of Al, X30Mn21, and X30Mn21+6.8Al powders.

Nanoindentation tests were carried out to examine the spherical indentation stress‐strain response at the regions of interest (ROIs) specified in **Figure** **1**. These tests were conducted on the same samples that had been previously prepared for microscopy. To determine mechanical properties, an Agilent G200 Nanoindenter (KLA Inc., Milpitas, CA, USA) was used, equipped with an XP head and a continuous stiffness measurement (CSM) module. The testing involved a diamond conical indenter with a tip radius of 100 µm. Indentations were applied at a constant nominal strain rate of 0.05 s⁻¹, calculated as the loading rate divided by the applied load, reaching a maximum depth of 800 nm. The CSM module operated with an oscillation frequency of 45 Hz and a displacement amplitude of 2 nm. A minimum of 10 indentations were performed on each individual ROI, totaling 240 tests across all samples.^[^ ^31^ ^]^

SEM images were used to confirm the exact locations of indentations. Post‐test imaging of residual indents via SEM provided further insights into the deformation mechanisms in the material (Figure S5, Supporting Information). The obtained load‐displacement data was converted into indentation stress‐strain curves following the analytical procedures outlined in the subsequent section (Figure S6, Supporting Information).

The analysis protocols used in this work are based on Hertz's theory,^[^ ^32^ ^]^ and have been presented and validated in prior literature.^[^ ^33^, ^34^, ^35^, ^36^, ^37^, ^38^, ^39^ ^]^ In these protocols, the indentation stress and the indentation strain are calculated as^[^ ^40^, ^41^ ^]^

(1) $$\sigma_{ind}=\frac{P}{\pi a^{2}}$$

(2) $$\varepsilon_{ind}=\frac{4}{3\pi }\;\frac{h_{t}}{a}$$

(3) $$a=\frac{S}{2E_{eff}}$$

where *P*, *h_t_*, *S*, *a*, and *E_eff_* denote the indentation load, the total indentation depth, the stiffness (from CSM), the contact radius, and the effective indentation modulus. *E_eff_* is estimated from the initial elastic loading segment of the curve, and assumed to be same for the entire loading history imposed on the sample.^[^ ^42^ ^]^ From each measured indentation stress‐strain curve, the indentation elastic modulus, the indentation yield strength, and the indentation work hardening rate were extracted. A 0.002 plastic strain offset was employed to identify the indentation yield strength. The indentation work hardening rate was extracted by fitting a line to the indentation stress‐strain plot from the indentation yield point up to 0.015 indentation plastic strain. Indentation techniques have already been utilized extensively in prior studies^[^ ^43^, ^44^, ^45^, ^46^, ^47^ ^]^ for the mechanical characterization of diverse material systems. One of their main advantages is that they allow studies of material responses using very small volumes. In recent work, we have demonstrated the benefits of the spherical indentation stress‐strain protocols described above for (i) the rapid exploration of PSP relationships in structural alloys,^[^ ^48^, ^49^, ^50^, ^51^ ^]^ and (ii) obtaining new physical insights into microscale deformation mechanisms in composite materials.^[^ ^33^, ^34^, ^35^, ^36^ ^]^ These newly developed protocols offer several key advantages: i) the ability to extract meaningful indentation stress‐strain curves that distinctly separate elastic and plastic regimes, ii) the use of initial loading segments instead of unloading segments, which are typically used in conventional protocols–‐this approach allows for significantly smaller indentation depths, thereby reducing the required material sample volume for reliable mechanical characterization, and iii) the flexibility to systematically adjust the sample volume probed during indentation experiments by employing indenter tips of varying radii. This enables primary indentation zones to be controlled within a range of ≈50 nm to 500 µm in most metallic alloy samples. The values obtained from these tests are summarized in Table S2 (Supporting Information).

#### 2.1.8 Data‐Driven Structure‐Property Relationship and Statistical Analysis

The resulting data frame described under Section 4.6 for building the structure‐property ML models consists of 18873 × 6 (rows × columns). Each row represents individual grains. Further information on the distribution of input features and on their correlation can be seen in supplementary Figure S7 and Table S3 (Supporting Information). The input vectors were further pre‐processed by normalization and randomized in order. The complete available experimental dataset was split into 70% train set and 30% test set.

Building the (micro)structure‐property relationship was formulated as regression task using the open‐source pycaret library.^[^ ^52^ ^]^ Highly correlated features were removed, and 10‐fold cross‐validation was used for model validation. Besides R^2^ values, pycaret provides further results on error metrics, e.g., by mean squared error (MSE), and mean absolute error (MAE). The trained and tested model was then further analyzed with SHAP measures using the Python‐based SHAP (SHapley Additive exPlanations) library.^[^ ^53^ ^]^ In contrast to other methods, SHAP values offer model‐agnostic interpretations of local and global feature contributions on model outputs. For further information on explainability of data models, we refer to previous publications.^[^ ^19^, ^54^ ^]^ So far, transferability of the model on other EHLA setups (different laser settings or powder nozzle types) is not directly possible. An ongoing study focuses on the possibility of transfer learning and the limitations to build a generalized ML model for the EHLA process. Python code was used to complete all pre‐processing and ML‐related tasks.

#### 2.1.9 Phase Diagram Calculations

Calculations of equilibrium phase diagrams were performed using Thermo‐Calc 2019b (Thermo‐Calc Software, Sweden) with the PrecHiMn‐4 thermodynamic database developed for HMnS.^[^ ^55^ ^]^

## 3 Results

### 3.1 EHLA Process and Specimen Preparation

Figure 1a illustrates the extreme high‐speed laser material application (EHLA) used to fabricate the specimens. The powder particles revealed a spheroidal shape and were sieved, and air separated to ensure a size distribution between 10–45 µm (Figure 1a,d). Figure 1b shows the deposited material of X30Mn21 on the substrate cylinder with a targeted variation in the aluminum content (from 0 to 10 wt.%), along the build direction (BD) of the specimen. Four different laser powers, from 2400 W up to 3000 W, were used to fabricate the specimens. The cross‐section of each of the deposited materials (for all samples) was analyzed using the protocol depicted in Figure 1c. The cross‐section is the optical micrograph of the EHLA specimen fabricated at 2400 W laser power, showing the 6 different layers of “specimens”, each specimen with varying Al content as we traverse from bottom to top. The specimens are differentiated using ROI 1 up to ROI 6. Subsequently, these nomenclatures are modified in terms of their measured and averaged Al concentration from SEM EDS. Each specimen with a specific Al content was ≈8 layers thick. The overlapped zones were identified using optical and SEM imaging, however, the individual layers with specific aluminum compositions were identified away from these overlapped zones shown as ROI 1 to ROI 6. Initial observations showed that the first layer of ROI‐1 specimen partially remixed with the substrate cylinder. A comparable examination was conducted on the specimens fabricated with 2600, 2800, and 3000 W, respectively. The melt pool geometry, depth, and other extensive analyses using optical microscopy will be discussed in the subsequent sections.

**Figure 2:**
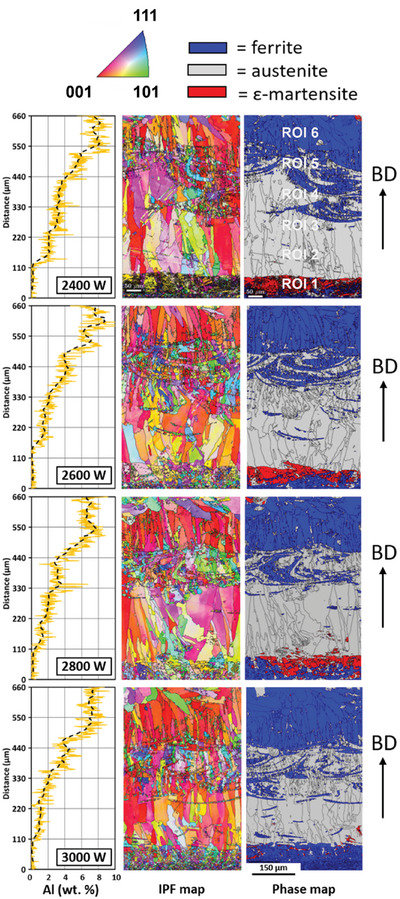
Integrated EBSD/EDS measurements along the build direction (BD) of six EHLA‐printed layers at four different laser powers. The Al content added to the base X30Mn21 steel was gradually increased from bottom to the top (left column) which changed microstructure and existing phases. Microstructure representation is using color‐coding according to the inverse pole figure (middle column) and to the phases present (right column) region.

Microstructure and chemistry analysis on EHLA specimens in Figure 2 presents the integrated EBSD and EDS measurements along BD of the six EHLA‐printed specimens at four different laser powers. The EDS measurements are presented as plots of the distance (along BD in mm) versus aluminum content (wt.%). IPF maps and the corresponding phase maps depicting ferrite (blue), austenite (grey), and ε− martensite (red) are shown. The EDS line scans were taken along the center of the ROI. The initial analysis was aimed at qualitative estimation of the amount of aluminum content within each of the specimens. The black dashed line shows the average of the Al content measured from the line scans. In general, the plots depict that measured Al content gradually increases from the base layer of X30Mn21 steel to ≈8 wt.% across all the samples. The yellow lines illustrate the local fluctuations in the Al concentration within each layer. Such a phenomenon is anticipated in rapid solidification processes due to solute redistribution and local phase transformations. The IPF maps (middle column) reveal the differences in grain size, along BD, for all the specimens. A significant intermixing of the base layers with the substrate material is also observed for all the specimens. The phase maps (right column) clearly show that the base layers are predominantly austenitic (with low Al content) grains that are columnar in nature. However, with increasing Al content along BD, there is a transition region with a mixed fcc austenite and bcc ferrite region. Additionally, from the IPF maps, these regions appear to be fine‐grained in nature. At higher Al contents, the microstructures are fully ferritic. Interestingly, the ROI‐1 regions for all the specimens exhibit a small fraction of ε−martensite. However, the change in the local chemistry due to the intermixing of the base layers of ROI‐1 with the substrate material makes it challenging to decipher the phase transformation trends in ROI‐1 layers. Hence, for the subsequent discussions, the ROI‐1 layers will not be considered. In addition, the ROI‐1 specimens reflect the base material of X30Mn21 alloy, which has been extensively analyzed in our prior LPBF^[^ ^24^ ^]^ and DED‐based studies.^[^ ^7^, ^25^ ^]^ These prior results serve as a baseline for understanding the results obtained in this study from the EHLA process with varying Al content (**Figure** **2**).

### 3.2 Chemical distribution within the Fabricated EHLA Specimens

**Figure 3:**
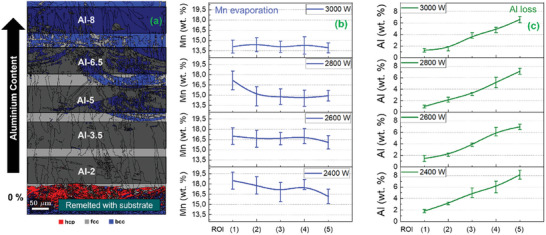
Panel (a) is the EBSD‐generated phase map of the sample produced at 2400 W with increasing Al concentration (target value of 2 to 10 wt.%). The map is divided into individual regions of interest (ROI) based on the Al content. The layer of X30Mn21 (with 0% Al) is not considered for the chemical analysis because of the intermixing with the low‐carbon steel substrate during solidification. Panels (b) and (c) are the Mn and Al contents in the individual ROIs depending on laser power and sample height by EDS measurements in the middle of every ROI. Vertical lines indicate error bars.

**Figure** **3**a presents the IPF image from the EBSD data for the specimens fabricated with 2400 W. **Figure** 3b,c present EDS plots showing the variation of Mn and Al content between the different ROIs, for specimens fabricated with all the EHLA laser powers. Each point on the plots is the Mn and Al content (wt.%) averaged from the EDS line scans across the transverse direction up to a distance of at least 150 µm of each ROI. This is further elucidated in Figure S1 (Supporting Information). For all the specimens produced, we observe Mn vaporization. This reduces its content in all specimens (for all input laser energies) below the targeted value of 21 wt.%. The elevated Mn vapor pressure compared to the other elements drives its evaporation, as observed in other LPBF processing of HMnS.^[^ ^7^, ^56^ ^]^ The lower Mn content leads to significant effects on the local stacking fault energy, phase stabilities, and the corresponding phase transformation kinetics.^[^ ^24^, ^57^, ^58^ ^]^ Since the measured average Al content in the individual ROIs is slightly lower than the targeted values, we rename the individual ROIs in terms of their measured Al contents, which are Al‐2, Al‐3.5, Al‐5, Al‐6.5, and Al‐8 respectively.

### 3.3 Grain Size and Phase Evolution

**Figure 4:**
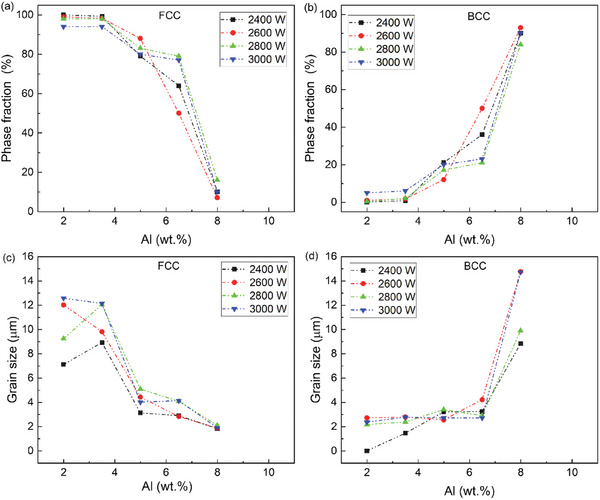
Plots showing the influence of Al content on the a,b) average phase fraction and c,d) average grain size evolution (evaluated from the EBSD data) of the individual FCC and the BCC phases for varying laser inputs (2400–3000 W).

**Figure** **4**a,b compares the evolution of the phase fractions for all the specimens and average grain size for all phases as a function of Al content. Both plots were extracted from the EBSD data for all the specimens. The evolution of the phase fractions for fcc and bcc phases follows the stability of the austenite and ferrite phases with increasing Al content.

The grain size evolution for the fcc and bcc phases are compared in Figure 4c,d. All the specimens show a significant refinement of the austenite grain size from Al‐2 to Al‐8 resulting from increasing phase transformations. The ferrite grain size is initially less than 3 µm for all the specimens, however, the grain size increases by at least 5 times for all the specimens. In addition, there is a significant local variation in the trends based on the processing condition. This microstructural control toward grain refinement and potential texture randomization by modifying the laser AM process parameters is possible in HMnS steels with targeted additions of Al. This has been exploited in previous works using both LPBF and DED techniques.^[^ ^7^, ^24^, ^25^, ^56^ ^]^ By virtue of modifying the solidification sequence, grain refinement of the fcc phase was possible, although dependent on the limited range of the liquid to fcc phase transformation and the stability of the fcc phase. Grain refinement of the FCC phase is driven by the interplay between thermodynamics and kinetics during solidification.^[^ ^59^, ^60^ ^]^ Processes like EHLA, with their rapid cooling and high‐temperature gradient,^[^ ^12^, ^61^ ^]^ provide conditions for promoting finer FCC grains. However, the extent of refinement is limited by the stability of the FCC phase, the liquid‐to‐FCC transformation range, and EHLA process parameters

The Al‐2 and Al‐3.5 specimens exhibit a minor fraction of bcc grains at the melt pool boundaries (also see 3). In specimens fabricated with 2400 W, the ferrite grains are less than 1 µm in size. Al‐5 and Al‐6.5 samples show a duplex microstructure with a significant refinement of fcc grains (nearly 50%) and larger bcc grains in the melt pool boundaries, specifically in Al‐6.5, for specimens fabricated with 2400 W (also see 3). Specimens of Al‐8 exhibited significant fcc grain refinement (2 µm) compared to the Al‐2 specimens, and a pronounced size of bcc grains particularly for samples fabricated with 2600 and 3000 W. Experimental and thermodynamic calculations have shown that in Al‐alloyed HMnS, the solid‐state transformation of delta bcc to fcc occurred during solidification cooling.^[^ ^56^ ^]^ This solid‐state phase transformation potentially hinders epitaxial growth, thereby promoting the nucleation and growth of fcc grains. This mechanism contributes to the grain refinement observed specifically in the fcc grains at higher Al contents.

The EHLA‐fabricated specimens show a dominant response to the evolution of grain size, grain morphology, and phase fraction evolution comparable to its LPBF and DED counterparts. The evolution of fine‐grained austenite‐ferrite duplex microstructure in the Al‐5 and Al‐6.5 specimens can be attributed to the transition from a fully fcc mode to fcc‐bcc mode of solidification.^[^ ^24^, ^56^, ^57^ ^]^ Al‐2 and Al‐3.5 samples were fully austenitic and comparable to samples produced via LBPF^[^ ^56^, ^62^ ^]^ and DED.^[^ ^7^ ^]^ The fcc‐bcc mode of solidification observed in Al‐5 is comparable to the observations made in LPBF‐processed X30Mn21 with 5 wt.% of Al.^[^ ^56^ ^]^ For Al‐6.5 and Al‐8, the stability of the bcc phase increases irrespective of the input energy of the laser (with the other EHLA parameters being constant) and the subsequent effects on cooling rate, the solidification mode is predominantly ferritic.

### 3.4 Effect of Melt Pool Characteristics and Phase Transformations in EHLA Specimens

**Figure 5:**
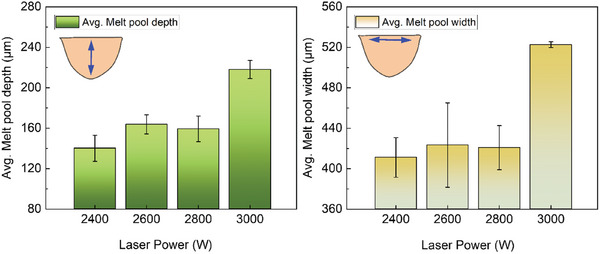
Bar graphs showing the variation of the average melt pool depth and width as a function of increasing input laser power during the EHLA process. All the measurements were performed on the topmost layer of the individual samples. Vertical lines indicate error bars.

**Figure** **5** shows the influence of the input laser power in the EHLA process on the melt pool geometries of all the specimens. The measurements were performed for the topmost layer of the specimens, which constitutes the ROI Al‐8. In general, the melt pool depth and width are the lowest for the 2400 W specimens. The melt pool depth increases by 16%, from an average of 150 µm at 2400 W to ≈175 µm for 2600 W specimens. Similarly, there is a 17% increase in the measured melt pool width, from 425 µm at 2400 W to ≈500 µm for 2600 W specimens. However, only a slight increase in the melt pool depth and width is observed as the laser power is further increased from 2600 until 3000 W. Generally, higher laser speeds coupled without pre‐heating of the substrate plate drive shallower and thinner melt pools, subsequently leading to higher cooling rates. However, deeper and wider melt pools are characteristic of lower laser scan speeds, leading to lower cooling rates.^[^ ^24^ ^]^

### 3.5 Microstructure‐Property Assessment using Nanoindentation

**Figure 6:**
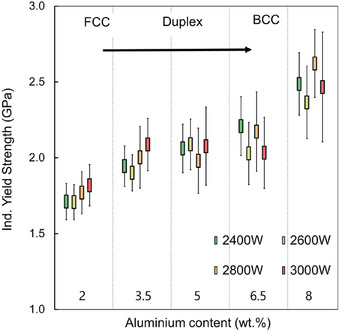
Dependence of indentation yield strength on the Al content (wt.%) and input laser power of EHLA process.

The indentation stress‐strain curves are extracted for all nanoindentation measurements. The SEM images of the indentation imprints after unloading at different laser powers and different Al content are in Figure S5 (Supporting Information). In addition, Figure S6 (Supporting Information) shows the series of indentation stress‐strain curves collected at the center of each ROI for all laser powers and Al contents. The indentation yield strength is defined as the indentation stress at 0.002 (offset) plastic indentation strain. The highlighted band represents the average indentation yield strength with one standard deviation. The indentation yield strength as a function of the Al content is plotted in **Figure** **6**.

In the EHLA‐fabricated specimens, the wide range of achieved microstructures with different alloy compositions and laser power allowed for achieving compositions with varying local scale mechanical properties. The indentation yield strength gradually increases with Al content up to 8 wt.%. Due to the larger melt pools induced by the EHLA process at 3000 W, the ferrite fraction of at least 2% was observed in the specimens. This contributed to the higher yield strength amidst all specimens of Al‐2. In addition, the secondary effect of the melt pool depth variation and the higher cooling rates (10^4^ K s^−1^) refines the parent austenite grains (PAG) due to large thermal gradient and slow growth rate. Studies have shown the prior austenite grain size at very high laser power (4000 W) was calculated at 8 µm.^[^ ^61^ ^]^ Hence EHLA solidification conditions can generate fine columnar PAGs that further contribute to the grain refinement. Furthermore, the general increase in the yield strength of the specimens can be attributed to the increasing content of the bcc phase coupled with solid solution strengthening by Al. The solute Al enhances the solid solution strengthening in HMnS (by ≈20 MPa/wt.%) due to its effect on lattice distortion and modulus misfit.^[^ ^63^ ^]^

### 3.6 Data‐Driven Structure‐Property Relationship

**Figure 7:**
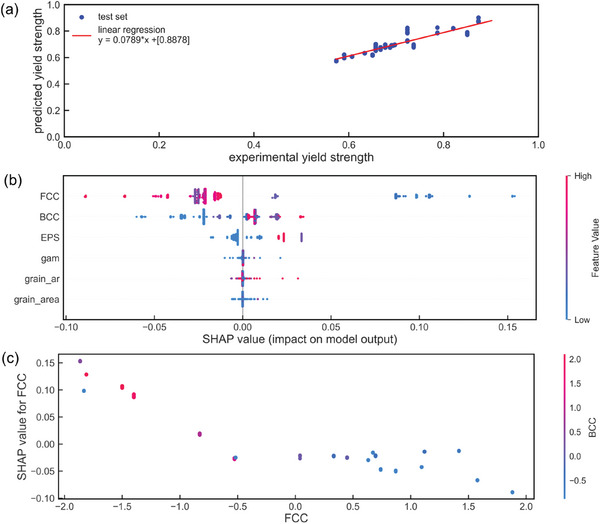
a) Regression plot on the actual and predicted yield strength (derived from indentation testing) on the test dataset, b) global feature importance reveals phase fractions as driving factors for the yield strength prediction, e.g. decreased fractions of austenite (FCC) result in higher SHAP values (high SHAP values (red color) indicate a higher probability for a material response with higher yield strength). b) Partial dependence of austenite (FCC) and fraction of ferrite (BCC) shows in tendency to lower SHAP values for higher values of FCC combined with lower values of BCC. EPS, grain_aspect ratio (ar), grain_area, and gam represent fraction of ε‐martensite, grain aspect‐ratio, and the area of grains, respectively.

To derive a fast yet interpretable relationship between (micro)structure and properties, machine learning (ML) based on the experimental data was used to generalize the SP relationship. Selected grain descriptors from EBSD data (section 0) were merged with measured phase fractions in a pandas data frame containing grain area, aspect ratio of grains (AR), grain average misorientation (GAM), austenite, ferrite, and ε‐martensite. The target variable derived from averaged nano hardness measurements (Section 2.5) represents a microscale property. In the following, this target variable will be denoted as yield strength, but referring to a microscale‐related yield strength derived by nanoindentation. The correlation with macroscopic mechanical behavior (yield strength) is evaluated in Section 3.3. After testing on previously unseen data, such an ML model allows interference for new values of the input variables without the necessity to run additional experiments. A non‐comprehensive parameter study was used to determine the model parameters of the artificial neural network as described in Section 2.8. Pycaret^[^ ^52^ ^]^ was used to identify the Gradient Boosting Regressor (GBR) as model with the lowest error metrics (e.g., mean average error, root mean squared error) and the highest R^2^ value of 0.89 and a mean squared error (MSE) of 0.0004, as seen in the parity plot shown in **Figure** **7**a indicating that the model predictions agreed well with the experimental data. Therefore, within the boundaries of the input dataset, the model has achieved a reasonable prediction of the yield strength of the alloys.

So far, the ML model lacks further information on the individual contributions of microstructural features on the yield strength. Therefore, a subsequent SHAP analysis was used to reveal further insights into individual and global contributions of microstructural features. SHAP values allow for quantitative evaluation of input feature contributions. A rather high or low data point (indicated by its index color) represents a positive (SHAP value > 0) or negative (SHAP value < 0) contribution to the output. For example, the greatest impact on the SHAP values, and therefore on an increase of yield strength, was identified by a low fraction of austenite marked in blue (Figure 7b). Features, describing the grain morphology appear more important than the grain average misorientation (GAM). Additionally, dependence plots using the SHAP library enable pair‐wise feature analysis on the yield strength (Figure 7c). The increase in the yield strength intensifies if low fractions of austenite are paired with higher fractions of ferrite.

## 4 Discussion

### 4.1 Effect of Local Chemical Heterogeneities on Phase Transformation Behavior

In Figure 3b the fluctuations in Mn content within the EHLA specimens are comparable to the LPBF‐produced specimens of comparable input energy densities.^[^ ^56^, ^57^ ^]^ However, the fluctuations are far less pronounced as compared to DED‐fabricated HMnS of similar composition.^[^ ^7^ ^]^ In addition, one observes significant heterogeneity of Al chemistry across the transverse direction, at each ROI and in all the specimens with varying Al content (Figures S1, S2, Supporting Information), due to the non‐equilibrium solidification. Coupling these process‐induced conditions with increasing Al content has a dual effect of acting as a stabilizer of the ferrite phase, increasing the SFE of the alloy, thereby, affecting the stability of the austenite phase. This creates a scenario where regions with low Al content and low SFE exhibit transformation‐induced plasticity (TRIP) during deformation (typically in tension). With subsequent increases in Al content, the deformation mode changes to twinning‐induced plasticity (TWIP) enhancing ductility and shifts finally to pure dislocation slip at high Al contents.^[^ ^7^, ^24^, ^25^ ^]^

In the Al‐2 specimen for the 2400 W EHLA sample, the Al content fluctuates between 1.2 to 2.5 wt.%. The strong heterogeneity in Al distribution presented in the EDS measurements in Figure 4, has a definitive influence on the phase transformation behavior at the local regions of the microstructure. This is particularly advantageous in this alloy design and screening approach. Al has several benefits amongst other bcc stabilizing solute elements. Addition of Al to Fe‐based alloys reduced the density of the alloy by 1.3% per wt.%, enhances solid‐solution strengthening in HMnS, delays hydrogen embrittlement in HMnS, and increases the SFE by 8 mJ m^−2^ per wt.%.^[^ ^23^, ^56^, ^64^ ^]^ More importantly, with targeted additions of Al to X30Mn21 and its subsequent partitioning within the local regions of the microstructure, we aim to tune the solidification sequence by stabilizing the bcc ferrite phase. The combined effects of Marangoni‐induced convection and increased interface velocities during the EHLA process can reduce the degree of micro segregation of Al to the interfaces, thereby enhancing ferrite formation. This phenomenon is relevant at the regions in the microstructure which exhibit a high degree of coarse grain formation, as the solidification induced partitioning behavior helps in achieving a more homogeneous ferrite microstructure.^[^ ^24^, ^59^ ^]^ This is observed in the plots of the phase fraction evolution with increasing Al content for all the EHLA fabricated samples. As the Al content increases, the phase composition (Figure 4a,b) of the EHLA‐printed specimen evolves from fully austenitic (Al‐2, Al‐3.5), to a mixed austenitic and ferritic microstructure (Al‐5, Al‐6.5), and subsequently to an almost fully ferritic microstructure (at Al‐8). This is consistent in specimens fabricated with 2400 and 2600 W. For specimens fabricated with 2800 and 3000 W, similar trends were quantified except in the Al‐2 condition, where a very small fraction (<2%) of ε−martensite is observed (Figure S3, Supporting Information). The formation of ε−martensite from austenite is thermally induced, possibly resulting due to the low SFE and destabilization of fcc phase because of higher loss of Mn.^[^ ^57^ ^]^ Formation of ε−martensite phase at the intradendritic regions in the as‐built microstructure of HMnS has been observed in LPBF‐^[^ ^56^ ^]^ and DED‐fabricated samples.^[^ ^7^ ^]^ However, with increasing Al content the immediate consequence is an increase in the SFE and higher driving force for the formation of the bcc phase, leading to the pronounced phase transition from a single‐phase fcc to a duplex microstructure containing austenitic and ferritic phases as the composition changes from Al‐3.5 until Al‐6.5. For all the Al‐8 specimens. Irrespective of the input EHLA laser power, the microstructure is predominantly ferritic with nearly 15% austenite phase content.

In HMnS, increasing the Al content coupled with reducing Mn content (due to evaporation) has a dual impact. First, it enhances the stabilization of the ferrite phase due to Al's strong ferrite‐forming tendency. Second, this change affects the grain growth behavior, as higher Al concentrations tend to refine grains by reducing the driving force for grain coarsening. Meanwhile, the reduction in Mn content, an austenite stabilizer, suppresses the formation of austenite, further promoting ferrite dominance. TEM analysis showed bcc grains decorated the prior melt pool boundaries, aided by the compositional heterogeneities, i.e., bcc grains were enriched in Al and depleted in Mn or vice versa for the fcc grains, and the alternating bcc‐fcc grains preferred to have a Kurdjumov‐Sachs orientation relationship.^[^ ^56^ ^]^ Since the cooling rate in the EHLA process is in the range of 10^4^ to 10^6^ K s^−1^, it would be a reasonable inference to use the understanding from existing studies in AM‐processed HMnS for discussing the microstructural evolution. The local chemical redistribution in Mn and Al content in all the EHLA specimens, due to microsegregation and phase transformations, affects the stability of the austenite. The comparison of the calculated equilibrium phase diagram of X30Mn21 with varying Al content is presented in Figure S4 (Supporting Information). The lowest and the highest input laser power of the EHLA process was selected to compare the effect of Mn and Al variation on the stability of the austenite in these respective specimens. For the 2400 W specimens, the average Mn content in the microstructure measured for all the Al‐2 until Al‐8 ROIs from EDS is 17.44 wt.%. The austenite phase was calculated to be stable until nearly 6.4 wt.% of Al. However, with further energy input at 3000 W, the Mn vaporization was exacerbated and the average Mn content in these specimens was reduced to 14.16 wt.%. Therefore, the stability of the austenite shifted to lower Al contents at 5.6 wt.%.

### 4.2 Melt pool Geometry Effects on Phase Transformation

For specimens fabricated with low Al contents (Al‐2 and Al‐3.5), the primary solidification mode is austenitic. However, higher laser input energy leads to lower cooling rates because of larger melt pools induced by the EHLA process. Therefore, this reduced cooling rate coupled with the redistribution of Al and Mn, provides a greater driving force and time for ferrite formation from austenite.^[^ ^7^, ^24^ ^]^ Therefore, we observe a ferrite fraction of at least 2% for specimens fabricated with 3000 W, which is the highest when compared with other specimens (see Figure 4). For the compositions between Al‐3.5 and Al‐6.5, the phase transformation is dictated by the redistribution of Mn and Al within the interdendritic and melt pool boundaries. At higher Al contents, the primary solidification mode is ferritic. However, for higher laser energy the melt pool size is larger, subsequently lowering the cooling rate. Hence there is more time for austenite formation. Therefore, at 8 wt.% Al, the amount of austenite is still significantly higher (between 7–16%) for all the specimens.

The melt pool depths reported here (Figure 5) are at least 60% higher than values reported for LPBF of HMnS.^[^ ^24^ ^]^ The remelting of the previous layers because of deeper laser penetration creates a higher probability for epitaxial growth, potentially strengthening the crystallographic texture. In addition, from the context of rapid solidification, equiaxed grain formation is driven by enhanced grain fragmentation and solute enrichment, both amplified by high cooling rates typical in processes like additive manufacturing.^[^ ^5^, ^59^ ^]^ However, due to the fcc‐bcc type of solidification for specimens with intermediate and higher Al content, the crystallographic texture can be weakened to a more isotropic random form. Kies et al. used a mathematical time‐dependent heat model to simulate the melt pool geometry of the DED process of HMnS + 1% Al, with 2 different laser spot sizes, and found that the melt pool dimensions were like the width of the laser beam.^[^ ^25^ ^]^ The laser beam diameter used in the present EHLA study is close to 3 mm. Therefore, the melt pool geometries measured in the present alloy design study using EHLA are between size range of LPBF and DED melt pool geometries. This further validates EHLA to be a potential tool that accommodates changes in cooling rate by modification of the process to meet DED and LPBF solidification conditions.

### 4.3 Transferring from EHLA to LPBF

Using the data‐driven structure‐property relationship, the prediction of the yield strength obtained using selected grain descriptors from EBSD data (Section 2.4) was merged with process‐related parameters that agreed well with the yield strength derived from nanoindentation measurements. To test the transferability of high‐throughput alloy design using EHLA and ML, LPBF samples using powder mixtures (X30Mn21 + x (Al)) at 120 W laser power were fabricated. This is the lowest input energy density of the LPBF process, comparable to the EHLA sample fabricated at 2400 W. In addition, the highest part density (min 99.5%) was achieved using these LPBF process parameters. The samples were fabricated at 2 different scan rotations, namely 0°, that mimics the EHLA process, and the other at 90° to evaluate the upper bound of the scan rotation. More details regarding the process parameters have been explained in the methods Section 2.2. Six different alloy powders of X30Mn21+ xAl were prepared using powder mixtures. The final Al composition of these alloys after LPBF fabrication varied between 0 to 6.8 wt.%. Further details of the Al content have been elucidated in the table in Table S1 (Supporting Information).

**Figure 8:**
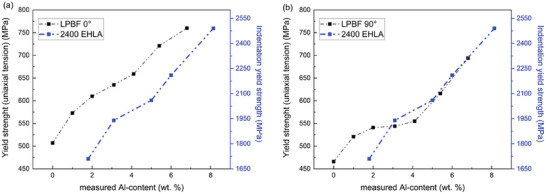
Plots comparing the yield strength obtained from the uniaxial tensile test of LPBF‐fabricated X30Mn21 and its powder blend derivates with increasing Al content vis‐à‐vis the yield strength obtained from nanoindentation of the EHLA‐fabricated alloys of X30Mn21 with targeted layer‐wise increase in Al content from 2 to 8 wt.%, fabricated using a laser power of 2400 W. The calculated yield strength values obtained via the nanoindentation experiments for the EHLA specimens are summarized in Table S1 (Supporting Information) in the supplementary. Tensile specimens with two different scanning rotations, 0 and 90°, were also used.

**Figure** **8** shows the plots of the yield strength of the LPBF‐produced specimens obtained from macroscopic tensile test, compared with the indentation yield strength of the EHLA specimens fabricated at 2400 W. Figure 8a shows the evolution of the yield strength from uniaxial tensile strength for LPBF samples with 0° of scan rotation between subsequent layers. For the EHLA‐produced samples, the trends in the evolution of the indentation yield strength are comparable to the macroscopic tensile yield strength of the LPBF samples. In terms of absolute values, the yield strength of the LPBF samples (fabricated with lower and upper scan rotation values) from macroscopic tensile tests is ≈ $\frac{1}{3}$ of the indentation yield strength of the EHLA specimens (fabricated on a rotational substrate). LPBF samples produced with a 90° scan rotation (Figure 8), the yield strength values are lower compared to the 0° scan rotation samples. Within the boundary condition of varying macroscopic properties such as texture, grain size distribution, and residual stress state of the samples, the evolution of the macroscopic tensile yield strength for LPBF specimens was found to agree well with the trends observed in the indentation yield strength of EHLA specimens. This excellent agreement cements the proof of concept that the EHLA‐based high‐throughput experiments render a combined synthesis tool to fabricate samples with LBPF conditions along with the flexibility of DED conditions.

### 4.4 Assessment of the Present Methodology

The current strategy to fabricate samples with the EHLA process enables high‐throughput screening of multiple compositions for the additive manufacturing process in a single specimen. The evaluation of the mechanical properties along with the microstructure studies for the EHLA specimens cater to understanding firsthand the primary effects of the composition fluctuations on the additively manufactured specimens. The average indentation yield strength can serve as a surrogate measure of the bulk yield strength as a function of the composition variation (compared in Figure 8) but does not account for the effects of defects, grain boundaries, texture, and residual stresses that evolve during the individual specimens from the LPBF and DED process. However, this method provides an enhanced solution to accelerate high‐throughput alloy screening to further evaluate specific alloy compositions using laser additive manufacturing.

Gradient Boosting Regressor as an ML model was selected as most accurate among more than 20 different models for learning the relationship between microstructural features and the material's yield strength (derived from microhardness). The generalizability of the model is limited to the bounds of the training and testing dataset and is therefore not valid beyond the minimum and maximum of the derived yield strength values. The current ML model is trained in data originating from an EHLA printing machine. Transferability of the model on other EHLA setups (different laser settings or powder nozzle types) is not directly possible. An ongoing study focuses on the possibility of transfer learning and the limitations to build a generalized ML model for the EHLA process.

These data‐driven microstructure‐property relationships complement and generalize insights from a cascade of relative labor‐intensive individual experiments. But predictions alone by the trained model are of back‐box‐type. Only with support of methods coming from the field of explainable artificial intelligence (XAI), as for example by using LIME^[^ ^65^ ^]^ or calculating Shapley values, individual microstructural feature contributions can be correlated quantitatively with mechanical properties (e.g., the material's yield strength). In contrast to other conventional methods for interpretability, calculating Shapley values via SHAP is model‐agnostic and therefore applicable to various types of ML models. However, it must be noted, that SHAP values can be based on feature space‐inherent biases and can therefore be misleading. For this reason, correlations found with SHAP cannot be seen as causal linkages. With further experiments, such correlations could be proven to be causal.

However, a critical assessment of actual requirements for new alloys for additive manufacturing commonly demands desired mechanical properties or defines properties for a minimum valuable product (MVP), and asks how to design the microstructure, or better how to set the manufacturing parameters to achieve these properties. Such requests are inverse‐oriented, while until now, (also in our approach) methods are applied forward‐directed (e.g., from scanning potential process‐parameter spaces toward measuring mechanical properties or part performances afterward). Invertible neural networks (INN)^[^ ^66^ ^]^ are more suitable to give direct answers to such inverse‐oriented tasks while revealing more than one parameter setting (rather distributions of parameter settings) to satisfy required properties. First studies on invertible approaches applied to microstructures can be found in refs. [67, 68, 69]

As an example, an SEM dataset was used in ref. [70] to inversely design new ferritic martensitic 9–12 wt.% Cr alloys based on the clustered latent space reduced by kernel PCA of encoded microstructures with a variational autoencoder (VAE) network. The first principal component of the PCA‐reduced latent space was interpreted as parameter for new alloy identification. Other INN‐based approaches to material optimization aim at optical and thermal properties,^[^ ^71^ ^]^ dual phase (DP) steels,^[^ ^67^ ^]^ and requirements of photovoltaics for microstructure generation.^[^ ^72^ ^]^ For the EHLA dataset of this study, an INN model could be used to discover possible process parameter combinations where microstructures or mechanical properties of interest serve as input data for the model. These studies can be seen as a proof of concept but commonly lack addressing physics‐based knowledge. This becomes necessary for scarce datasets, a common characteristic for datasets in the field of material science. Possible adjustments of more powerful INNs consider variations in the loss functions, e.g. by adding terms to comply with realistic microstructure characteristics. Applying INNs on our EHLA dataset is part of our ongoing research.

The EHLA process for developing high‐manganese steels exhibits potential for economic savings and environmental sustainability. The current high‐throughput screening process reduces powder usage, material waste, and energy consumption, lower harmful fumes and byproducts, and lead times for alloy design for LPBF and DED processes. Furthermore, the selected HMnS alloys are durable and lightweight materials (due to their high specific strength) that are sustainable materials due to the high abundance of Fe and Mn compared to critical elements such as Co and Ni. With additions of Al, these lightweight components are used in downstream industries, such as automotive and renewable energy sectors which further improves fuel and energy efficiency.^[^ ^73^, ^74^ ^]^ The present EHLA alloy design concept of tuning local phase transformation behavior due to changing AM solidification conditions and the subsequent high‐throughput characterization and data analysis can be translated to all materials systems to design new materials for additive manufacturing.

## 5 Conclusion

Combined high‐throughput sample synthesis and alloy characterization were used to explore novel alloy compositions, their corresponding microstructure evolution, and mechanical properties in additively manufactured (AM) advanced high‐strength steel. Application of Extreme High Laser Application (EHLA) for the production of 24 individual alloy conditions with varying chemistry and/or processing history within a single specimen. The process‐structure‐property (P‐S‐P) relationships were revealed experimentally by large‐area EBSD analysis and nanoindentation stress‐strain protocols. The generated data served as a basis to derive a reliable machine learning (ML)–based P‐S‐P surrogate model. The following conclusions can be drawn:

- The employed methodology is a powerful toolset for AM‐specific alloy development as it allows for screening of additively manufactured states with strongly varying chemical composition and processing conditions. However, precise control of in‐situ alloyed element fractions, remelted volume, and related material mixing in the remelted region must be considered.
- The selected alloy is a model system, that is flexible in terms of phase stabilities because of the varying Al content. Therefore, during the EHLA synthesis, this promoted a wide range of achieved microstructures allowing for tailoring of the mechanical properties. The increased Al contents promoted solid solution strengthening in the austenitic conditions, grain refinement, and multi‐phase strengthening in the duplex conditions, and further strengthening due to ferrite stabilization in the ferritic condition.
- Combining open‐source libraries in Python allowed building satisfactory data models to generalize the relationship between microstructural features with mechanical properties with sufficient accuracy, but more importantly with explainable feature contributions.

The excellent agreement in trends between the mechanical properties of various alloys fabricated using LPBF and EHLA specimens (with a scale factor of 1/3) establishes EHLA to be a suitable impersonator to fabricate materials under LPBF conditions with the flexibility of incorporating DED conditions in a single high‐throughput alloy synthesis experiment.

## Conflict of Interest

The authors declare no conflict of interest.

## Author Contributions

A.H. and C.H. wrote the original draft; P.K., A.H., and C.H. conceived the idea, designed and performed the experiments; S.K. and P.K. performed nanoindentation experiments; A.H. and P.K. performed the data analysis and interpretation; A.K. and S.K. performed nanoindentation studies; M.A. performed the machine learning simulations; C.H. supervised the project; M.A., S.K., A.K., P.K., and S.K. revised and edited the manuscript. All authors discussed the results and finalized the manuscript.

## Supporting information



Supporting Information

## Data Availability

The data that support the findings of this study are available from the corresponding author upon reasonable request.
