# Supplementary material for: High‐Speed 3D Printing Coupled with Machine Learning to Accelerate Alloy Development for Additive Manufacturing
Source: Adv Sci (Weinh). 2025 Mar 7;12(17):2414880. doi: 10.1002/advs.202414880 (PMC12061307; doi:10.1002/advs.202414880)
Supplement: Supplementary file 1 — Supporting Information [file ADVS-12-2414880-s001.docx]

# Supplementary information

| **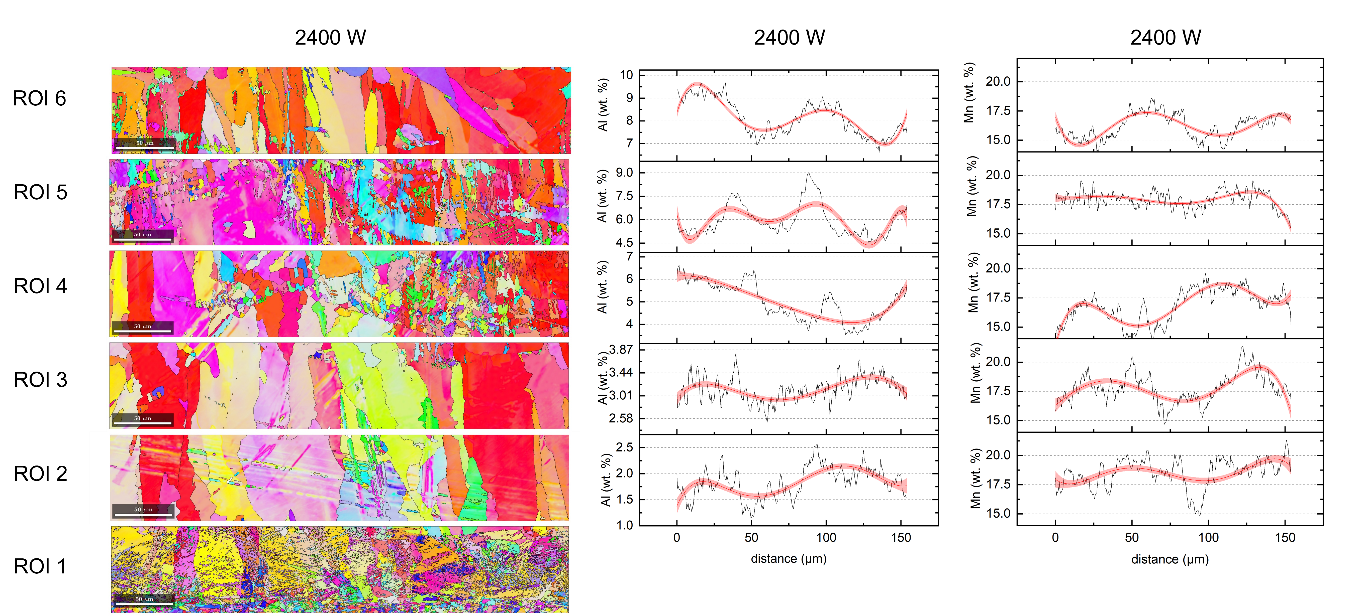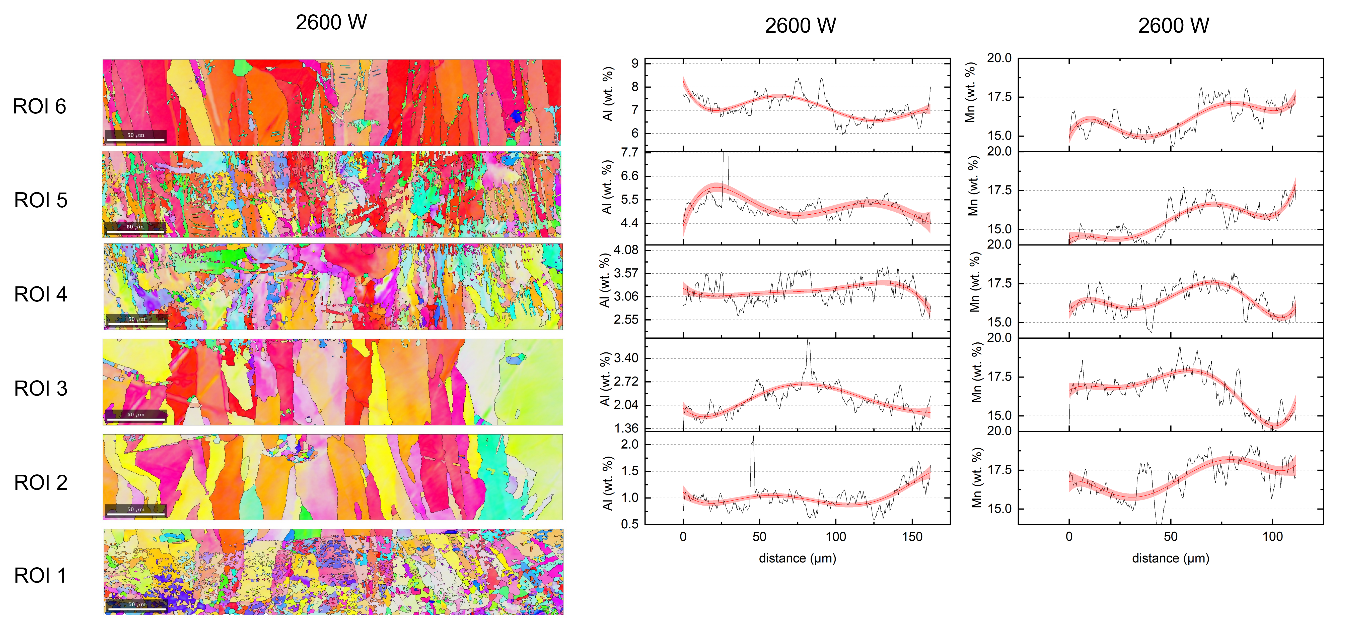**  Figure S1: EBSD IPF maps of the different ROIs of the EHLA produced X30Mn21 sample with increasing Al content and varying laser power (2400 W and 26000 W). The corresponding EDS line scans of Al and Mn across each layer, along the transverse direction (contd..). |
| --- |

| 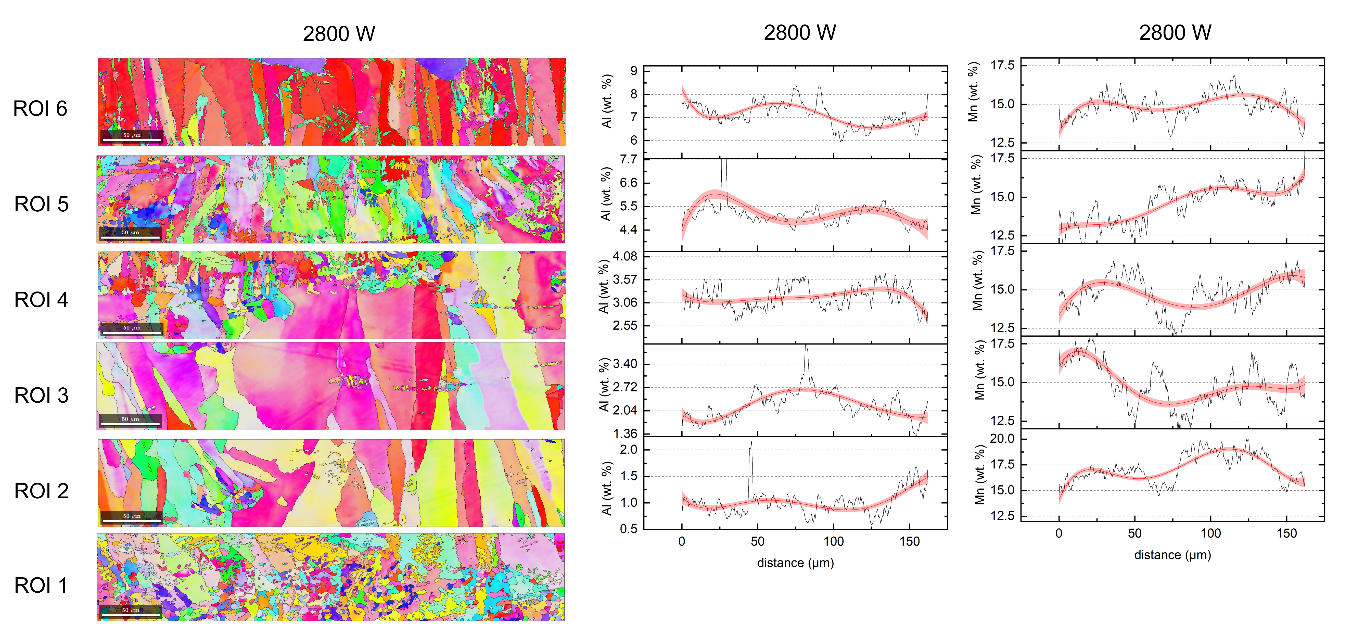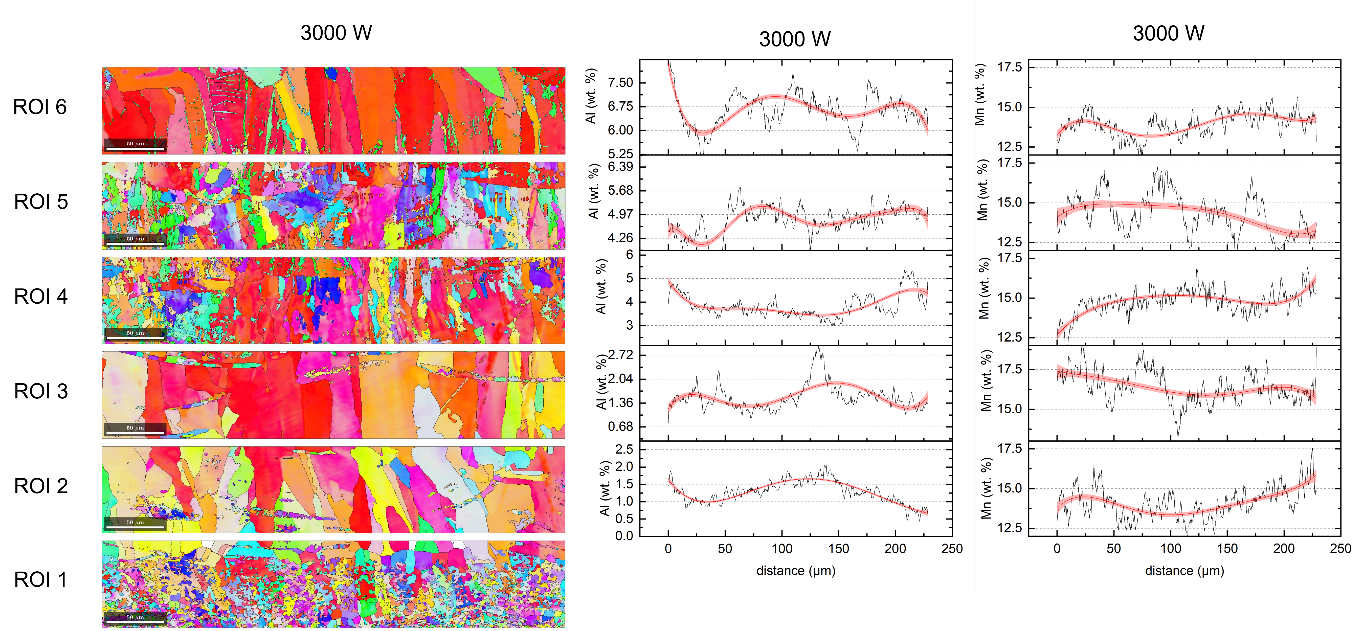  Figure S2: EBSD IPF maps of the different ROIs of the EHLA produced X30Mn21 sample at 2800 W and 3000 W laser power with increasing Al content . The corresponding EDS line scans of Al and Mn across each layer, along the transverse direction). |
| --- |

| 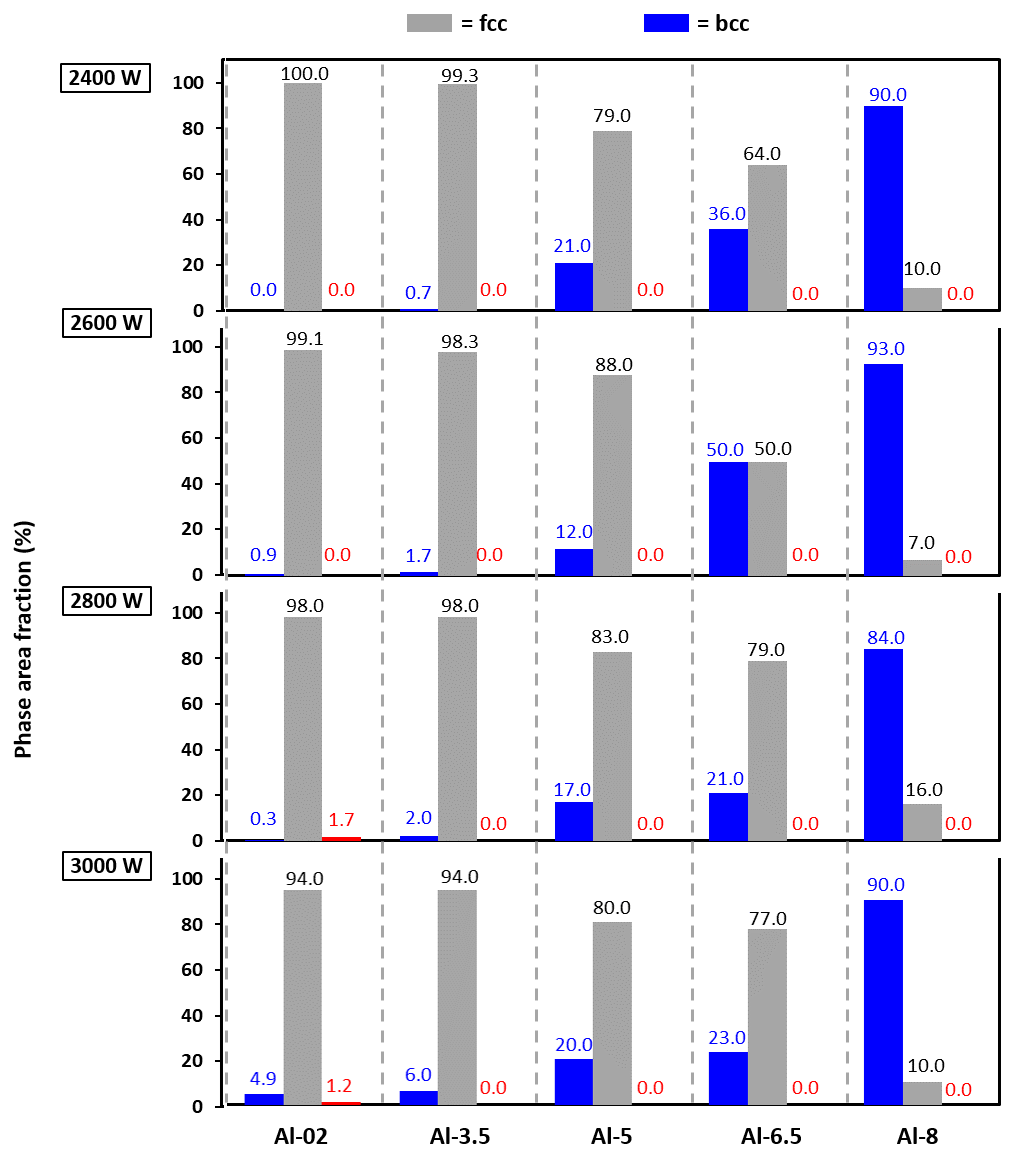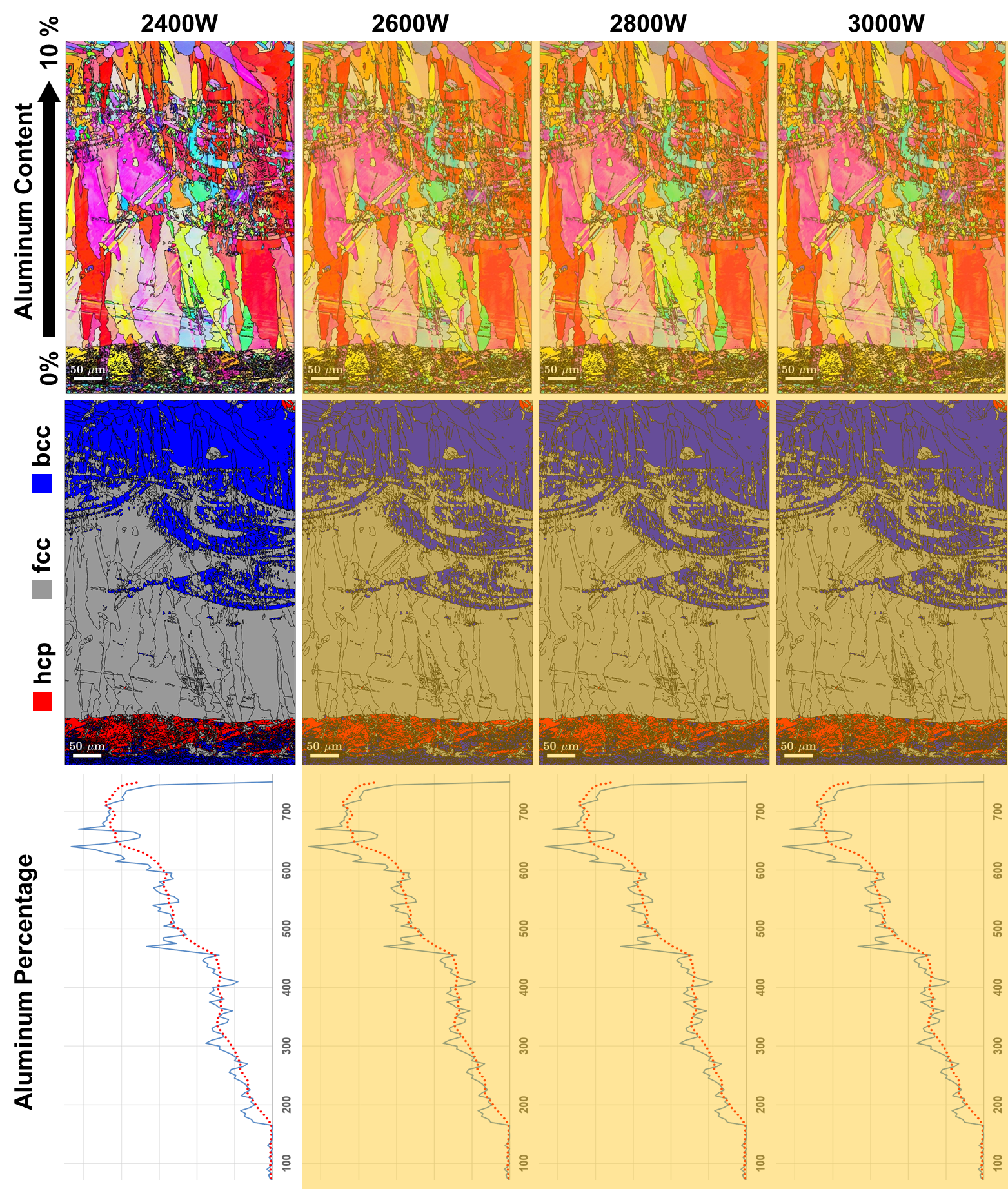 Figure S3: The bar graphs show the area fractions of each phase measured from each ROI of the EBSD data analysed for varying laser powers (2400 W – 3000 W) and different average Al content (2 – 8 wt.%) measured in the individual ROIs. |
| --- |

| **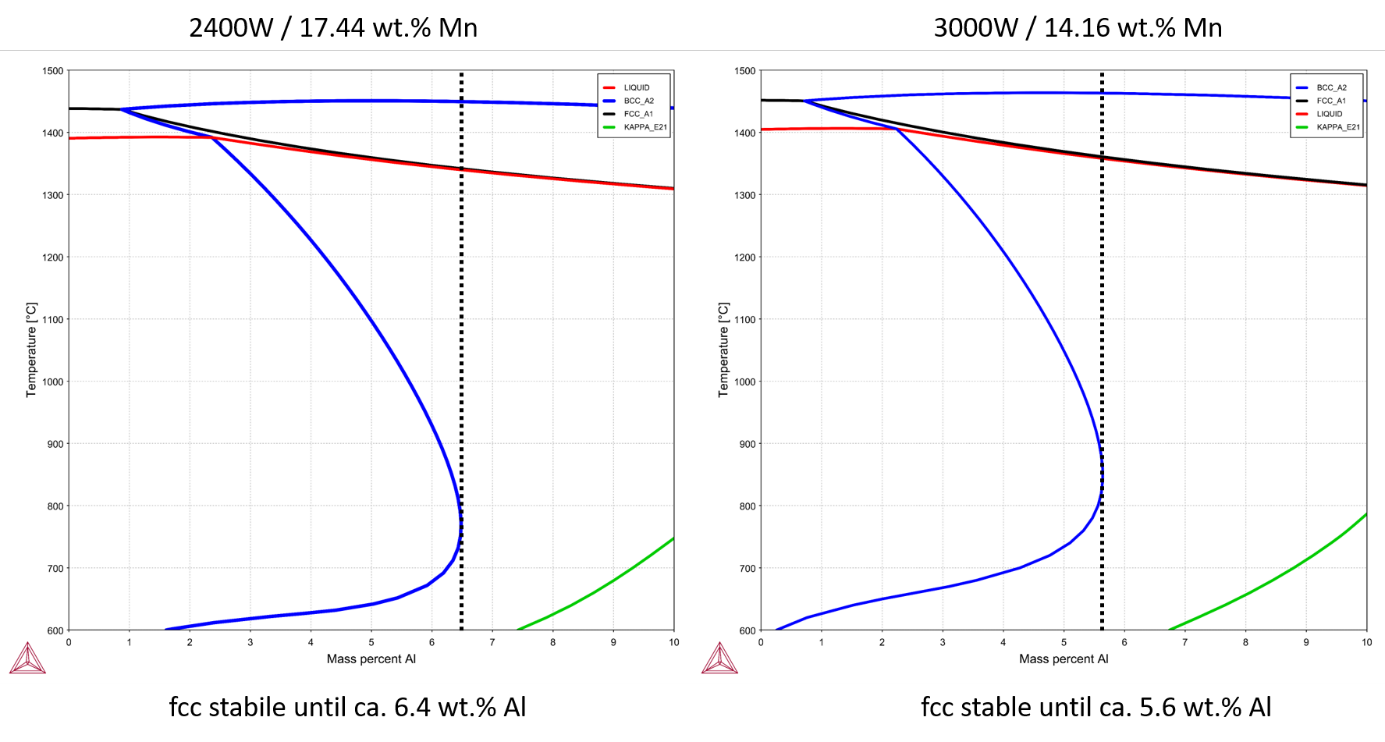**  Figure S4: Comparison of calculated equilibrium phase diagrams of X30MnAl21-x(Al), with varying Al content, considering the measured average Mn content after the EHLA process with 2400W and 3000W laser power. Process-induced Mn vaporisation reduces the fcc phase stability for higher laser power conditions. |
| --- |

| **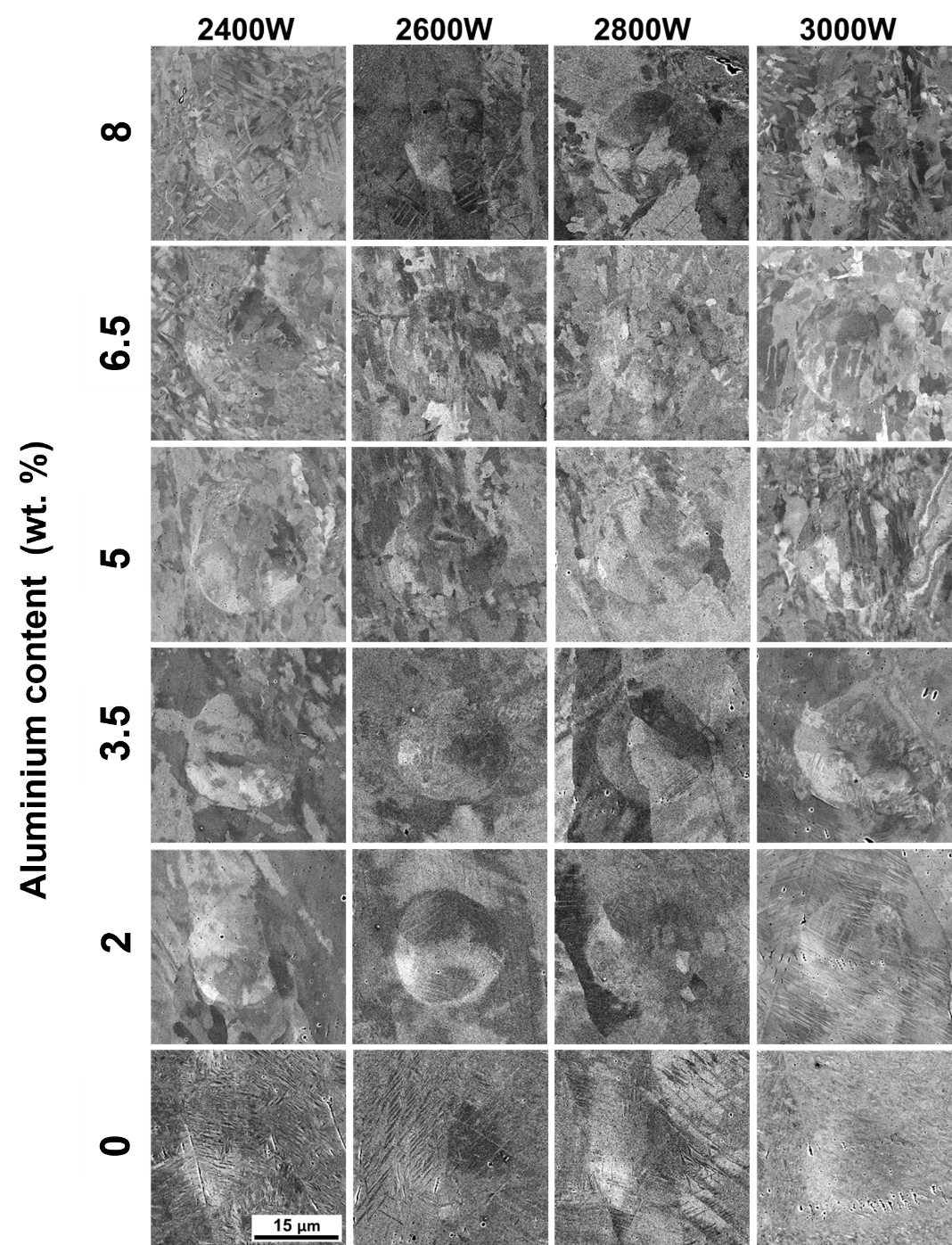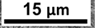**    Figure S5: SEM images of the indentation imprints after unloading at different laser power and different Al contents. |
| --- |

| 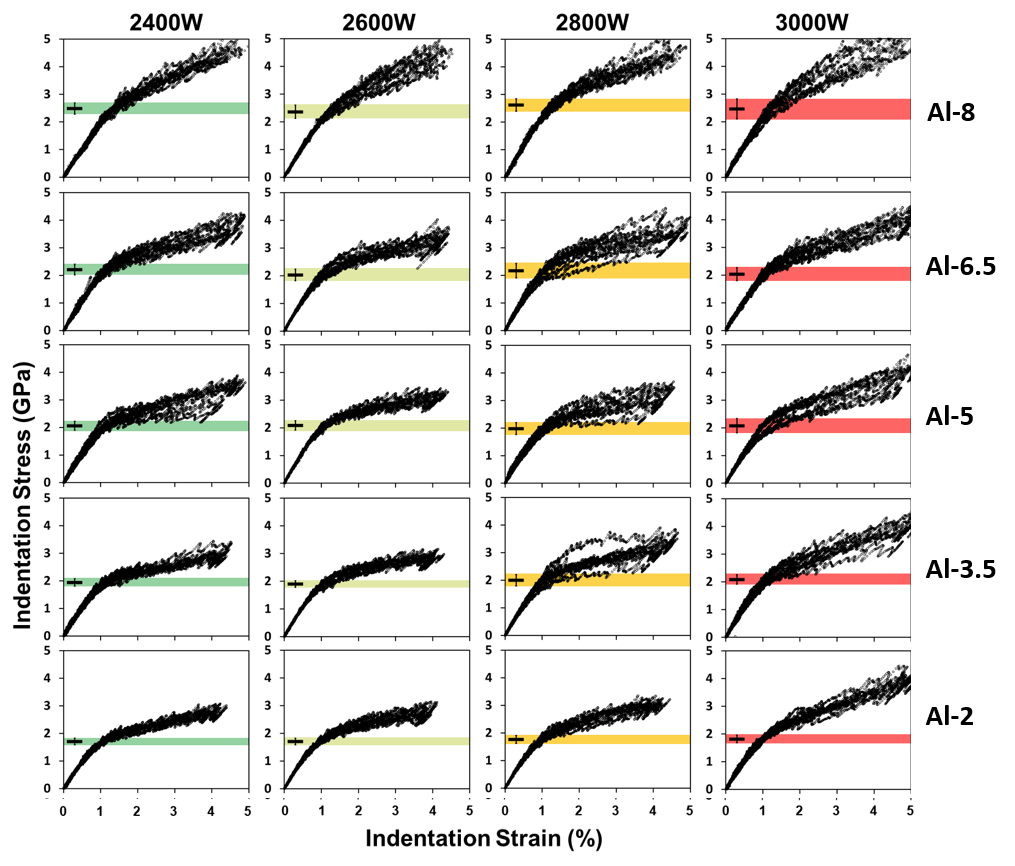    Figure S6: Multiple indentation stress-strain curves collected at the centre of each ROI for all laser powers and Al contents. The highlighted band represents the average indentation yield strength with one standard deviation. |
| --- |


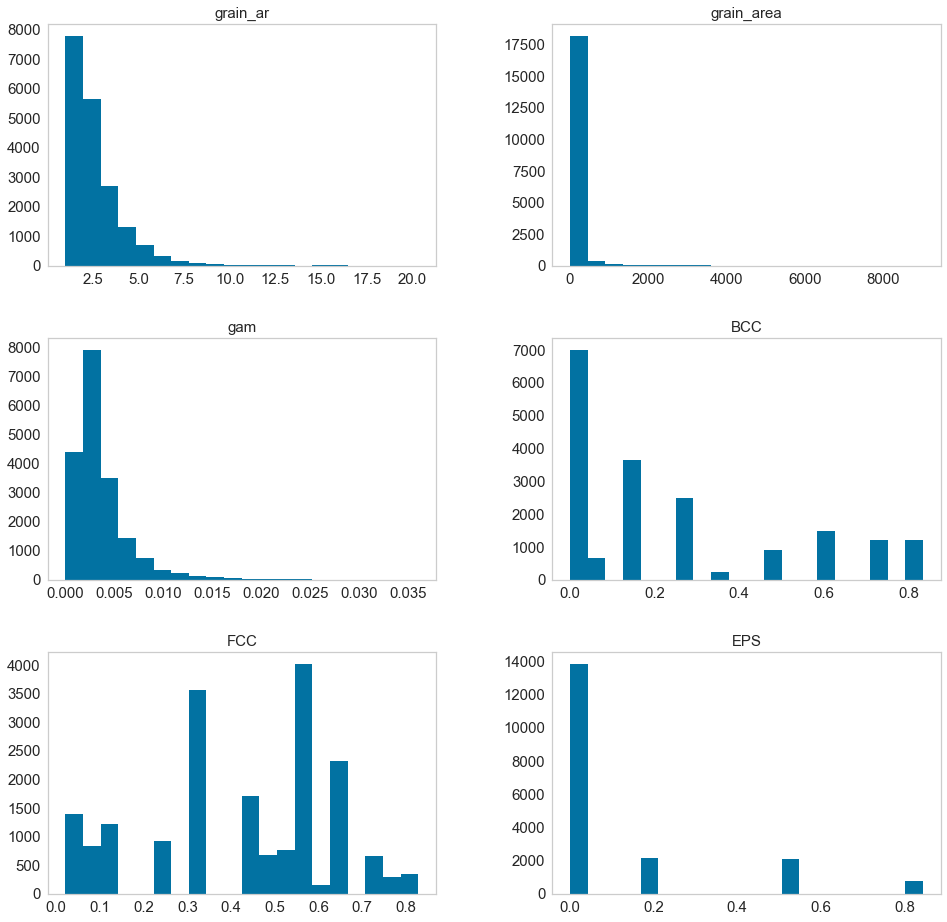


Figure S7: Distribution of input features used for building a data-driven structure-property relationship.

| Table S1: The yield strength values from tensile tests of LPBF-fabricated X30Mn21+ xAl for different scan rotations and their comparison with the indentation yield strength of the EHLA-fabricated samples.   \| **Al-wt. %** \| **LPBF 0°** \| **LPBF 90°** \| **Al-wt. %** \| **2400 EHLA** \| **Al-wt. %** \| **2600 EHLA** \| **Al-wt. %** \| **2800 EHLA** \| **Al-wt. %** \| **3000 EHLA** \| \| --- \| --- \| --- \| --- \| --- \| --- \| --- \| --- \| --- \| --- \| --- \| \| 0 \| 507 \| 466 \|  \|  \|  \|  \|  \|  \|  \|  \| \| 1 \| 573 \| 521 \|  \|  \| 1.5 \| 1710 \| 1 \| 1770 \| 1.3 \| 1820 \| \| 2 \| 610 \| 541 \| 1.8 \| 1710 \| 2.1 \| 1900 \| 1.2 \| 2000 \| 1.5 \| 2090 \| \| 3.1 \| 635 \| 544 \| 3.1 \| 1940 \| 3.84 \| 2090 \| 3.2 \| 1980 \| 3.9 \| 2080 \| \| 4.1 \| 659 \| 555 \| 5 \| 2060 \| 6.1 \| 2030 \| 5.2 \| 2170 \| 4.8 \| 2030 \| \| 5.4 \| 721 \| 616 \| 6 \| 2210 \| 7 \| 2360 \| 7.2 \| 2620 \| 6.6 \| 2470 \| \| 6.8 \| 760 \| 694 \| 8.14 \| 2490 \|  \|  \|  \|  \|  \|  \| |
| --- | --- | --- | --- | --- | --- | --- | --- | --- | --- | --- | --- | --- | --- | --- | --- | --- | --- | --- | --- | --- | --- | --- | --- | --- | --- | --- | --- | --- | --- | --- | --- | --- | --- | --- | --- | --- | --- | --- | --- | --- | --- | --- | --- | --- | --- | --- | --- | --- | --- | --- | --- | --- | --- | --- | --- | --- | --- | --- | --- | --- | --- | --- | --- | --- | --- | --- | --- | --- | --- | --- | --- | --- | --- | --- | --- | --- | --- | --- | --- | --- | --- | --- | --- | --- | --- | --- | --- | --- |

| Table S2: Young’s modulus, indentation yield strength, contact diameter at yield, and indentation work-hardening-rate of the individual conditions including errors (standard deviation).   \| **Laser Power (W)** \| **X30Mn22-xxAl** \|  \| **Young's Modulus (GPa)** \|  \| **Ind. Yield Strength (GPa)** \|  \| **Contact Diameter at Yield (μm)** \|  \| **Ind. Work Hardening Rate (GPa)** \| \| --- \| --- \| --- \| --- \| --- \| --- \| --- \| --- \| --- \| --- \| \| **2400** \| 2 \|  \| 182.4 ± 6.9 \|  \| 1.71 ± 0.12 \|  \| 4.8 ± 1.2 \|  \| 32.6 ± 8.6 \| \| 3.5 \|  \| 182.6 ± 8.6 \|  \| 1.94 ± 0.13 \|  \| 3.9 ± 1.2 \|  \| 26.0 ± 9.2 \| \| 5 \|  \| 190.7 ± 13.6 \|  \| 2.06 ± 0.16 \|  \| 4.1 ± 1.1 \|  \| 37.2 ± 10.6 \| \| 6.5 \|  \| 195.4 ± 13.6 \|  \| 2.21 ± 0.19 \|  \| 4.7 ± 1.2 \|  \| 40.8 ± 8.2 \| \| 8 \|  \| 184.1 ± 10.2 \|  \| 2.49 ± 0.21 \|  \| 4.9 ± 1.5 \|  \| 65.3 ± 5.1 \| \|  \|  \|  \|  \|  \|  \|  \|  \|  \|  \| \| **2600** \| 2 \|  \| 195.4 ± 8.5 \|  \| 1.71 ± 0.12 \|  \| 3.8 ± 1.4 \|  \| 34.5 ± 7.0 \| \| 3.5 \|  \| 200.2 ± 3.8 \|  \| 1.90 ± 0.12 \|  \| 2.8 ± 0.6 \|  \| 32.1 ± 12.4 \| \| 5 \|  \| 208.6 ± 5.7 \|  \| 2.09 ± 0.17 \|  \| 4.1 ± 1.5 \|  \| 35.5 ± 9.9 \| \| 6.5 \|  \| 208.4 ± 9.6 \|  \| 2.03 ± 0.21 \|  \| 3.4 ± 1.5 \|  \| 38.9 ± 13.1 \| \| 8 \|  \| 203.3 ± 10.4 \|  \| 2.36 ± 0.24 \|  \| 4.2 ± 1.5 \|  \| 65.3 ± 5.1 \| \|  \|  \|  \|  \|  \|  \|  \|  \|  \|  \| \| **2800** \| 2 \|  \| 198.2 ± 5.8 \|  \| 1.77 ± 0.14 \|  \| 5.1 ± 1.6 \|  \| 42.2 ± 8.7 \| \| 3.5 \|  \| 207.0 ± 11.4 \|  \| 2.00 ± 0.20 \|  \| 2.9 ± 0.7 \|  \| 36.7 ± 13.3 \| \| 5 \|  \| 200.5 ± 11.9 \|  \| 1.98 ± 0.21 \|  \| 3.2 ± 1.1 \|  \| 33.5 ± 16.8 \| \| 6.5 \|  \| 216.7 ± 10.0 \|  \| 2.17 ± 0.26 \|  \| 3.2 ± 0.9 \|  \| 37.9 ± 19.3 \| \| 8 \|  \| 201.7 ± 9.8 \|  \| 2.62 ± 0.22 \|  \| 3.7 ± 1.6 \|  \| 49.7 ± 12.3 \| \|  \|  \|  \|  \|  \|  \|  \|  \|  \|  \| \| **3000** \| 2 \|  \| 196.2 ± 7.8 \|  \| 1.82 ± 0.14 \|  \| 3.4 ± 0.8 \|  \| 47.6 ± 15.7 \| \| 3.5 \|  \| 199.5 ± 12.7 \|  \| 2.09 ± 0.17 \|  \| 3.5 ± 0.9 \|  \| 47.1 ± 9.4 \| \| 5 \|  \| 196.8 ± 12.8 \|  \| 2.08 ± 0.26 \|  \| 3.7 ± 0.9 \|  \| 44.0 ± 11.7 \| \| 6.5 \|  \| 201.9 ± 9.2 \|  \| 2.03 ± 0.23 \|  \| 3.1 ± 0.6 \|  \| 45.0 ± 13.2 \| \| 8 \|  \| 204.7 ± 6.3 \|  \| 2.47 ± 0.36 \|  \| 3.3 ± 0.7 \|  \| 66.1 ± 18.7 \| |
| --- | --- | --- | --- | --- | --- | --- | --- | --- | --- | --- | --- | --- | --- | --- | --- | --- | --- | --- | --- | --- | --- | --- | --- | --- | --- | --- | --- | --- | --- | --- | --- | --- | --- | --- | --- | --- | --- | --- | --- | --- | --- | --- | --- | --- | --- | --- | --- | --- | --- | --- | --- | --- | --- | --- | --- | --- | --- | --- | --- | --- | --- | --- | --- | --- | --- | --- | --- | --- | --- | --- | --- | --- | --- | --- | --- | --- | --- | --- | --- | --- | --- | --- | --- | --- | --- | --- | --- | --- | --- | --- | --- | --- | --- | --- | --- | --- | --- | --- | --- | --- | --- | --- | --- | --- | --- | --- | --- | --- | --- | --- | --- | --- | --- | --- | --- | --- | --- | --- | --- | --- | --- | --- | --- | --- | --- | --- | --- | --- | --- | --- | --- | --- | --- | --- | --- | --- | --- | --- | --- | --- | --- | --- | --- | --- | --- | --- | --- | --- | --- | --- | --- | --- | --- | --- | --- | --- | --- | --- | --- | --- | --- | --- | --- | --- | --- | --- | --- | --- | --- | --- | --- | --- | --- | --- | --- | --- | --- | --- | --- | --- | --- | --- | --- | --- | --- | --- | --- | --- | --- | --- | --- | --- | --- | --- | --- | --- | --- | --- | --- | --- | --- | --- | --- | --- | --- | --- | --- | --- | --- | --- | --- | --- | --- | --- | --- | --- | --- | --- | --- | --- | --- | --- | --- | --- |

Table S3: Correlation matrix of input features for building a data-driven structure-property relationship.

|  | grain_ar | grain_area | gam | BCC | FCC | EPS |
| --- | --- | --- | --- | --- | --- | --- |
| grain_ar | 1.000 | 0.004 | -0.032 | 0.102 | -0.113 | 0.000 |
| grain_area | 0.004 | 1.000 | -0.007 | -0.0546 | 0.048 | 0.015 |
| Gam | -0.032 | -0.007 | 1.000 | -0.116 | 0.118 | 0.037 |
| BCC | 0.102 | -0.0546 | -0.116 | 1.000 | -0.626 | -0.456 |
| FCC | -0.113 | 0.0480 | 0.118 | -0.626 | 1.000 | -0.348 |
| EPS | 0.000 | 0.0148 | 0.037 | -0.456 | -0.347 | 1.000 |
